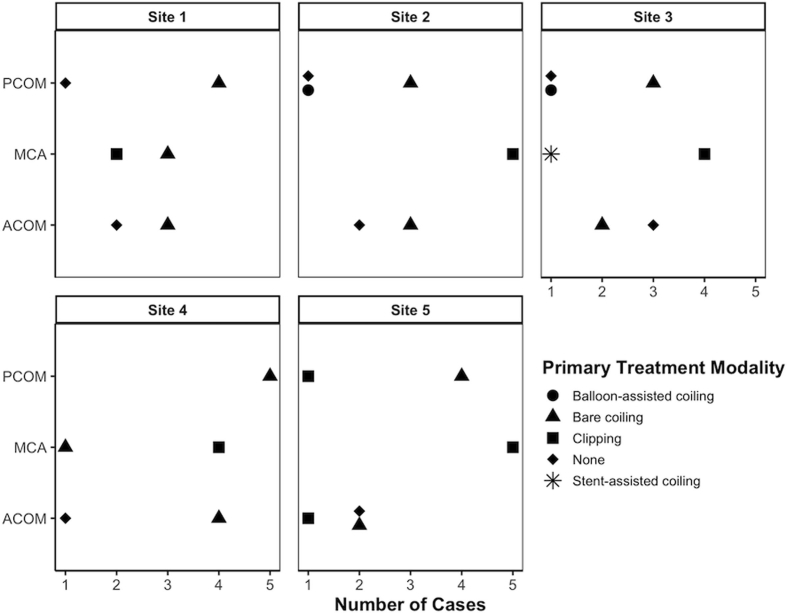# Treatment variation in acute management of patients with aneurysmal subarachnoid hemorrhage: a multicenter case vignette study

**DOI:** 10.1016/j.bas.2026.106071

**Published:** 2026-05-05

**Authors:** Rick J.G. Vreeburg, Daan de Groot, Harssh Verdan Bandral, Dagmar Verbaan, Ruben Dammers, Roel H.L. Haeren, Jeroen H.D. Boogaarts, Philippine B. van Wijngaarden, Mervyn D.I. Vergouwen, Thomas A. van Essen, Jeroen van Dijck, Wouter A. Moojen, SPARTA study investigators, Rene Aquarius, Karmen Aslankurt, Harssh Verdan Bandral, Rene van den Berg, Hieronymus D. Boogaarts, Bert A. Coert, Ruben Dammers, Jeroen T.J.M. van Dijck, Rahman Fakhry, Roel H.L. Haeren, Alexander Hamming, Wouter A. Moojen, Friso Mulder, Sacha Onstenk, Yasmin Sadigh, Hanna Schenck, Ewout W. Steyerberg, Dagmar Verbaan, Mervyn D.I. Vergouwen, Rick J.G. Vreeburg, Philippine B. van Wijngaarden

**Affiliations:** 8 Department of Neurosurgery, Radboud University Medical Center, Nijmegen, The Netherlands; 9 Department of Neurosurgery, Maastricht University Medical Center, Maastricht, The Netherlands; 10 Amsterdam University Medical Centers, Location AMC, Department of Neurosurgery, Amsterdam, The Netherlands; 11 Department of Neuro-radiology, Academic Medical Center Amsterdam, The Netherlands; 12 Department of Neurosurgery, Erasmus MC Stroke Center and Center for Complex Microvascular Surgery, Erasmus MC University Medical Centre, Rotterdam, The Netherlands; 13 University Neurosurgical Center Holland, LUMC, HMC and HAGA, Leiden and The Hague, The Netherlands; 14 Department of Biomedical Data Sciences, University Medical Center Utrecht, Utrecht, The Netherlands; 15 Amsterdam Neuroscience, Neurovascular Disorders, Amsterdam, The Netherlands; 16 Department of Neurology and Neurosurgery, Utrecht University Medical Center, UMC Utrecht Brain Center, Utrecht University, Utrecht, The Netherlands; 1 University Neurosurgical Center Holland, LUMC, HMC and HAGA, Leiden and The Hague, The Netherlands; 2 Amsterdam University Medical Centers, Location AMC, Department of Neurosurgery, Amsterdam, The Netherlands; 3 Amsterdam Neuroscience, Neurovascular Disorders, Amsterdam, The Netherlands; 4 Department of Neurosurgery, Erasmus MC Stroke Center and Center for Complex Microvascular Surgery, Erasmus MC University Medical Centre, Rotterdam, The Netherlands; 5 Department of Neurosurgery, Maastricht University Medical Center, Maastricht, The Netherlands; 6 Department of Neurosurgery, Radboud University Medical Center, Nijmegen, The Netherlands; 7 Department of Neurology and Neurosurgery, Utrecht University Medical Center, UMC Brain Center, Utrecht University, Utrecht, The Netherlands

**Keywords:** Subarachnoid hemorrhage, Endovascular, Clipping, Treatment variation, Drainage

## Abstract

**Background:**

Although a landmark randomized controlled trial (RCT) compared clipping with coiling in aneurysmal subarachnoid hemorrhage (aSAH), real-world practice remains heterogeneous. Advances in endovascular and microsurgical techniques, complex or borderline cases not well represented in trials, and differences in institutional expertise likely contribute to between-center variation. Understanding these treatment preferences is critical for identifying potential unwarranted variation and designing comparative effectiveness research that reflects decision-making.

**Methods:**

A multicenter case-vignette study was conducted across five Dutch tertiary referral centers for aSAH (n = 16 specialists) between May and October 2025. Fifteen real-world aSAH vignettes, representing diverse clinical and radiological profiles, were systematically presented during live meetings of the local neurovascular multidisciplinary teams (MDT), including neurologists, neurosurgeons, and interventional-radiologists. For each case, MDTs proposed an aneurysm treatment strategy. Between-center variation was quantified using Fleiss’ κ with 95% confidence intervals (CI).

**Results:**

Of 15 case vignettes, agreement on aneurysm treatment strategy among at least four of five centers was reached in 12 cases (80%). Regarding treatment modality, at least four of five centers opted for a similar modality in 10 of 11 cases (91%). The proportion of clipping versus any endovascular treatment option ranged between centers from 2/12 aneurysm treatments proposals (17%) to 8/13 proposals (62%), resulting in moderate agreement (κ = 0.49, 95% CI 0.31-0.68) for between-center variation.

**Conclusion:**

There is between-center variation in treatment strategies for ruptured intracranial aneurysms across Dutch tertiary referral centers for aSAH in identical case vignettes. These case-mix independent, center-level preferences may be leveraged for comparative effectiveness research.

## Highlights

• In a multicenter case-vignette study of identical aneurysmal subarachnoid hemorrhage scenarios, substantial between-center variation in aneurysm treatment modality persisted.
• The proportion of microsurgical clipping ranged from 17% to 62% across tertiary referral centers, resulting in only moderate between-center agreement.
• These case-mix–independent institutional preferences provide a pragmatic foundation for comparative effectiveness research when randomized trials are difficult to conduct.

## 1 Background

The annual incidence of aneurysmal subarachnoid hemorrhage (aSAH) is estimated to be approximately 6.1 per 100,000 people (Etminan et al., 2019; Hughes et al., 2018; de Rooij et al., 2007). Overall mortality is estimated to be up to 50% of the aSAH cases; among survivors, roughly half to two-thirds regain functional independence at 1 year (Asikainen et al., 2023; Lovelock et al., 2010; Nieuwkamp et al., 2009; Registry, 2022; Rinkel and Algra, 2011). Because aSAH frequently affects individuals in mid-life, the resulting long-term disability contributes significantly to socioeconomic burden (Rivero-Arias et al., 2010).

The acute onset of aSAH, severe neurological manifestation and the heterogeneity of the affected population complicate clinical decision-making in aneurysm treatment (Mooij, 2001; Hoh et al., 2023; Nieuwkamp et al., 2014). In the Netherlands, aneurysm treatment decisions are typically made within a multidisciplinary team setting (Neifert et al., 2021/06; Ran et al., 2023). Current practice and guidelines have been substantially influenced by results from the International Subarachnoid Aneurysm Trial (ISAT), with coiling considered the preferred treatment in cases of clinical equipoise (Molyneux, 2002; McDougall et al., 2012; Spetzler et al., 2015). However, long-term superiority of endovascular treatment over microsurgical clip reconstruction, forthwith referred to as clipping, remains debated, as ISAT primarily included aSAH patients in good clinical condition with anterior circulation aneurysms, and most screened patients were ultimately treated outside the trial.

The proportion of endovascular treatment versus clipping varies significantly across tertiary referral centers for aSAH, indicative of potential treatment preferences between each center (Neurochirurgie NVv; Lagares et al., 2015; de Winkel et al., 2021). Such variation may reflect differences in institutional expertise or case-mix. The extent of these potential treatment preferences is currently unclear. Understanding such treatment preferences may inform future improvement of aSAH guidelines and comparative effectiveness research (de Winkel et al., 2021; Hamming et al., 2024). In particular, clear center-level preferences may function as instrumental variables to investigate causal associations between treatment strategies and outcomes (Cnossen et al., 2018; van Essen et al., 2022/07; Baiocchi et al., 2014).

The aim of the current study was to identify and quantify treatment preferences across Dutch tertiary referral centers.

## 2 Methods

### 2.1 Study design and setting

A multicenter case vignette study was conducted between May 2025 and October 2025 across five of the nine Dutch tertiary referral centers for aSAH (Amsterdam University Medical Centers [AUMC], Haaglanden Medical Center [HMC], Maastricht University Medical Center+ [MUMC], Radboud University Medical Center [RUMC], and University Medical Center Utrecht [UMCU]).

### 2.2 Case selection

Fifteen case vignettes were selected from the Study on Prognosis of Acutely Ruptured Intracranial Aneurysms (SPARTA) study based on variability among patients regarding age, clinical condition at presentation, medical history, and aneurysm characteristics (Molyneux, 2002; Hamming et al., 2024; Evans et al., 2015; Plaum et al., 2024). To ensure a representative sample, we sampled 5 anterior communicating artery (ACOM), 5 middle cerebral artery (MCA), and 5 posterior communicating artery (PCOM) aneurysms with differing clinical profiles and patient characteristics. Posterior circulation aneurysms were not included during the design of the study, as their management is often more strongly weighted toward endovascular treatment. Therefore, we did not expect sufficient treatment variation in primary treatment modality to inform the study objective. All cases were discussed in a fixed sequence (i.e., equal during all meetings) during live multidisciplinary meetings at each of the five vascular centers. Thus, in total, there were 75 proposed treatment strategies.

### 2.3 Multidisciplinary team meetings

Each meeting sought to simulate the day-to-day multidisciplinary decision-making process within that participating center. The composition of each multidisciplinary team (MDT), therefore, varied but included medical specialties (neurology, neurosurgery, [interventional-]radiology) that are usually involved in the decision-making process for aneurysm treatment within each center. For each clinician, specialty, age, gender, and years of post-residency experience were recorded to describe the composition of the MDTs.

During each case discussion, the clinical characteristics and imaging (non-contrast CT, CT-angiography and, if available, diagnostic angiography [DSA]) would be provided to the multidisciplinary team (Table 1, Supplemental Table 1). Cases were presented by the same member of the research team using a structured, fixed format, but the discussion that followed regarding the optimal treatment strategies were conducted according to local MDT practices. No formal time limits per case were imposed, but no center exceeded a total of 90 min for all 15 cases. Interaction between MDT members was allowed and encouraged to reflect real-world clinical decision-making. The MDTs were asked to define a treatment strategy for each case, namely performing primary treatment for aneurysm repair (versus [initial] conservative management or treatment limiting decision making) and choice of treatment modality (clipping, coiling or other) (Table 2). In total, there were fifteen case vignettes in five centers, equaling 75 total treatment strategies. The MDTs were also required to provide the reasoning for their choice of primary treatment modality (Table 2 & Supplemental Material 1). Consensus was defined as complete between-center agreement (5 of 5 centers) proposing the same treatment decision, whereas disagreement was defined as any instance in which fewer than 5 of 5 centers proposed the same treatment decision.

**Table 1:** Clinical and imaging characteristics of the 15 case vignettes.

Case 1	**Patient:** 63-year-old female, living independently at home with spouse. Per-acute headache today. No loss of consciousness or neurological deficits. **History:** COPD, no medication **Neurological exam:** E4M6V5, WFNS I, H&H I **NCCT:** SAH, no IVH. CT-A/DSA: left-sided saccular PCOM aneurysm, 8 mm, no other aneurysms.
**Case 2**	**Patient:** 34-year-old female, living independently at home with spouse. Per-acute headache yesterday. No loss of consciousness or neurological deficits. **History:** none, no medication **Neurological exam:** E4M6V5, WFNS I, H&H II **NCCT:** SAH, no IVH. **CT-A/DSA:** right-sided saccular MCA aneurysm, 8 mm, no other aneurysms.
**Case 3**	**Patient:** 64-year-old male, living independently at home with spouse. 2 weeks of mild headache. Suddenly collapsed while waiting outside for a traffic light. EMT personnel noticed incoherent speech and a right-sided decrease in strength. **History:** TIA (>10 years ago) for which use of Ascal 1 80 mg tablet daily. **Neurological exam:** E4M6V4, right-sided hypotonic hemiplegia, WFNS III, H&H IV. **NCCT:** SAH, large left-sided lobar parieto-temporal hemorrhaging and a left-sided fronto-parietal acute subdural hematoma. **CT-A/DSA:** left-sided saccular MCA (bifurcation) aneurysm, 5 mm, no other aneurysms.
**Case 4**	**Patient:** 61-year-old female, living independently at home with spouse. Per-acute headache today during driving (car). Experienced light headedness and hyperesthesia in both arms. **History:** Hypertension, no medication. Active smoker. **Neurological exam:** E3M6V5, WFNS II, H&H II **NCCT:** SAH with IVH. **CT-A/DSA:** right-sided saccular PCOM aneurysm, 2 mm, no other aneurysms.
**Case 5**	**Patient:** 86-year-old female, living independently at home as a widow. Found today in bed by family. Last seen well three days ago, no symptoms at that time. The patient has a known do-not-resuscitate registration from two years ago. **History:** none, no medication **Neurological exam:** E2M4V1, WFNS IV, H&H V **NCCT:** SAH with IVH. **CT-A/DSA:** saccular ACOM aneurysm, 5 mm, no other aneurysms.
**Case 6**	**Patient:** 75-year-old male, living independently at home as a widow. Found this morning by brother on the bathroom floor in his home. Last seen well 24 h ago, no symptoms at that time. **History:** atrial fibrillation, hypertension and a mitral valve replacement >10 years ago. Uses Apixaban, dosage unknown. **Neurological exam:** E1M2V1, two non-reactive isocore pupils. No evident sign of lateralization. WFNS V, H&H V **NCCT:** SAH with IVH. **CT-A/DSA:** saccular ACOM aneurysm, 4 mm, no other aneurysms.
**Case 7**	**Patient:** 52-year-old female, living independently at home. Per-acute headache with loss of consciousness. Midnight presentation (01:00) in peripheral hospital with E1M3V2 (WFNS V, H&H V) where she is intubated and transported to the treating vascular center. **History:** none, no medication. Active smoker (25 packyears). **Neurological exam:** E1M1Vtube at first presentation in treating center (02:00). After initial conservative management until the morning (06:30) she scores E3M4Vtube. **NCCT:** SAH with IVH. **CT-A/DSA:** saccular ACOM aneurysm, 8 mm, no other aneurysms.
**Case 8**	**Patient:** 63-year-old female, living independently at home with spouse. Found in bed after she did not show up to work. EMT personnel noticed E1M4V1 (WFNS V, H&H V), after which she was intubated and sedated and brought to the treating center. **History:** depressive symptoms, citalopram use. **Neurological exam:** E1M1Vtube (intubated and sedated), isocore, non-reactive and small pupils. **NCCT:** SAH with IVH. **CT-A/DSA:** right-sided saccular PCOM aneurysm, 4 mm, no other aneurysms.
**Case 9**	**Patient:** 80-year-old female, living independently at home with spouse. Per-acute headache seven days ago, after which consistent mild headache symptoms, GP felt “something was off” and send the patient to the ER. **History:** none, no medication **Neurological exam:** E4M6V5, WFNS I, H&H I **NCCT:** SAH, no IVH. **CT-A/DSA:** saccular ACOM aneurysm, 3 mm, no other aneurysms.
**Case 10**	**Patient:** 62-year-old female, living independently at home with spouse. Per-acute headache today, no loss of consciousness or neurological deficits. **History:** hypertension, medication usage unknown. **Neurological exam:** E3M6V4, WFNS II, H&H III **NCCT:** SAH with IVH. **CT-A/DSA:** right-sided saccular MCA (distal M2) aneurysm, 3-4 mm, no other aneurysms.
**Case 11**	**Patient:** 71-year-old female, living independently at home as a widow. Per-acute headache this morning without loss of consciousness or neurological deficits. **History:** well-regulated hypertension, medication unknown. Active smoker (50 packyears). **Neurological exam:** E4M6V5, no neurological deficits, WFNS I, H&H I **NCCT:** SAH, no IVH **CT-A/DSA:** left-sided saccular PCOM aneurysm, 6 mm, smaller contralateral (right-sided) saccular PCOM aneurysm (1 mm) and ACOM aneurysm (1 mm).
**Case 12**	**Patient:** 65-year-old female, living independently at home with spouse. Per-acute headache last night with nausea and emesis. Spouse could not wake the patient adequately this morning, since then the patient has been somnolent. **History:** well-regulated hypertension and myocardial infarction (>10 years ago) for which use of Ascal 1 80 mg tablet daily. **Neurological exam:** not able speak coherently, only speaks words (no sentences), E3M6V4, WFNS II, H&H II **NCCT:** SAH, no IVH **CT-A/DSA:** right-sided saccular PCOM aneurysm, 11m, no other aneurysms.
**Case 13**	**Patient:** 84-year-old female, living independently at home as a widow. Per-acute headache today without loss of consciousness and no neurological deficits. **History:** suboptimal regulated hypertension and type II diabetes. Medication usage unknown. **Neurological exam:** E4M6V5, WFNS I, H&H I **NCCT:** SAH, no IVH **CT-A/DSA:** right-sided saccular MCA (trifurcation) aneurysm, 7 mm, no other aneurysms.
**Case 14**	**Patient:** 76-year-old female, living independently at home as a widow. Per-acute headache today without loss of consciousness and no neurological deficits. **History:** Coronary Artery Bypass Grafting (five years ago) and suspected Transient Ischemic Attack (six years ago) without rest symptoms. Uses 1 tablet of 75 mg clopidogrel daily. Active smoker (30 pack years). **Neurological exam:** E4M6V5, WFNS I, H&H I **NCCT:** SAH, no IVH. **CT-A/DSA:** saccular ACOM aneurysm, 3 mm, no other aneurysms.
**Case 15**	**Patient:** 41-year-old female, living independently at home with spouse. Per-acute headache two days ago without loss of consciousness and no neurological deficits. Today, again per-acute headache with nausea and emesis. **History:** multiple myocardial infarctions for which multiple percutaneous coronary interventions (two years ago, five years ago, six years ago, nine years ago). Recent in-stent thrombosis (last month) for which she now uses Ticagrelor (two 90 mg tablets daily) and Ascal (one 80 mg tablet daily). **Neurological exam:** E4M6V5, WFNS I, H&H I **NCCT:** SAH with IVH. **CT-A/DSA:** right-sided saccular MCA (bifurcation) aneurysm, 15 mm, no other aneurysms.

*Abbreviations:* ACOM, anterior communicating artery; CT-A, computed tomography angiography; DSA, digital subtraction angiography; EMT, emergency medical technician; EMV, eye, motor and verbal component of the Glasgow Coma Scale; ER, emergency room; H&H, Hunt and Hess grading system; IVH, intraventricular hemorrhage; MCA, middle cerebral artery; mm, millimeter; NCCT, non-contrast computed tomography; PCOM, posterior communicating artery; SAH, subarachnoid hemorrhage; TIA, transient ischemic attack; WFNS, World Federation of Neurosurgical Societies grading system.

**Table 2:** Questions and responses to the case vignettes across all centers (MDT).

Case	1	2	3	4	5	6	7	8	9	10	11	12	13	14	15
Primary Treatment (%)															
Clipping	0 (0)	4 (80)	4 (80)	0 (0)	0 (0)	0 (0)	1 (20)	0 (0)	0 (0)	5 (100)	0 (0)	1 (20)	4 (80)	0 (0)	3 (60)
Bare coiling	5 (100)	1 (20)	1 (20)	4 (80)	1 (20)	0 (0)	3 (60)	2 (40)	5 (100)	0 (0)	4 (80)	4 (80)	1 (20)	5 (100)	1 (20)
Stent-assisted coiling	0 (0)	0 (0)	0 (0)	0 (0)	0 (0)	0 (0)	0 (0)	0 (0)	0 (0)	0 (0)	0 (0)	0 (0)	0 (0)	0 (0)	1 (20)
Balloon-assisted coiling	0 (0)	0 (0)	0 (0)	1 (20)	0 (0)	0 (0)	0 (0)	0 (0)	0 (0)	0 (0)	1 (20)	0 (0)	0 (0)	0 (0)	0 (0)
None (treatment limiting or initial conservative management)	0 (0)	0 (0)	0 (0)	0 (0)	4 (80)	5 (100)	1 (20)	3 (60)	0 (0)	0 (0)	0 (0)	0 (0)	0 (0)	0 (0)	0 (0)
Reasoning primary treatment^a^ (%)															
Neurological condition	1 (7.1)	1 (7.7)	2 (13.3)	2 (15.4)	0 (0)	0 (0)	1 (7.7)	1 (11.1)	3 (21.4)	1 (6.7)	2 (13.3)	2 (14.3)	1 (9.1)	1 (6.7)	0 (0)
Standard treatment of the hospital	0 (0)	1 (7.7)	2 (13.3)	1 (7.7)	1 (16.7)	0 (0)	1 (7.7)	1 (11.1)	1 (7.1)	1 (6.7)	1 (6.7)	1 (7.1)	0 (0)	1 (6.7)	0 (0)
Patient's age	1 (7.1)	3 (23.1)	0 (0)	0 (0)	1 (16.7)	0 (0)	0 (0)	0 (0)	2 (14.3)	0 (0)	1 (6.7)	1 (7.1)	1 (9.1)	3 (20)	3 (23.1)
Presence of comorbidities	0 (0)	0 (0)	1 (6.7)	0 (0)	0 (0)	0 (0)	0 (0)	0 (0)	0 (0)	0 (0)	0 (0)	0 (0)	0 (0)	0 (0)	2 (15.4)
Time between ictus and arrival in treating center	0 (0)	0 (0)	0 (0)	0 (0)	0 (0)	0 (0)	0 (0)	0 (0)	2 (14.3)	0 (0)	0 (0)	0 (0)	0 (0)	0 (0)	0 (0)
Use of anticoagulants	0 (0)	0 (0)	0 (0)	0 (0)	0 (0)	0 (0)	0 (0)	0 (0)	0 (0)	0 (0)	0 (0)	0 (0)	0 (0)	1 (6.7)	2 (15.4)
Aneurysm configuration	5 (35.7)	1 (7.7)	1 (6.7)	4 (30.8)	0 (0)	0 (0)	4 (30.8)	2 (22.2)	5 (35.7)	4 (26.7)	5 (33.3)	4 (28.6)	4 (36.4)	5 (33.3)	2 (15.4)
Aneurysm size	2 (14.3)	1 (7.7)	0 (0)	2 (15.4)	0 (0)	0 (0)	1 (7.7)	1 (11.1)	0 (0)	0 (0)	1 (6.7)	1 (7.1)	0 (0)	1 (6.7)	1 (7.7)
Aneurysm location	3 (21.4)	4 (30.8)	4 (26.7)	2 (15.4)	0 (0)	0 (0)	3 (23.1)	1 (11.1)	1 (7.1)	5 (33.3)	2 (13.3)	3 (21.4)	3 (27.3)	2 (13.3)	3 (23.1)
Concomitant hematoma evacuation	0 (0)	0 (0)	5 (33.3)	0 (0)	0 (0)	0 (0)	0 (0)	0 (0)	0 (0)	4 (26.7)	0 (0)	0 (0)	0 (0)	0 (0)	0 (0)
Other	2 (14.3)	2 (15.4)	0 (0)	2 (15.4)	0 (0)	0 (0)	2 (15.4)	0 (0)	0 (0)	0 (0)	3 (20)	2 (14.3)	2 (18.2)	1 (6.7)	0 (0)
Not applicable (no primary treatment)	0 (0)	0 (0)	0 (0)	0 (0)	4 (66.7)	5 (100)	1 (7.7)	3 (33.3)	0 (0)	0 (0)	0 (0)	0 (0)	0 (0)	0 (0)	0 (0)
Would you be willing to leave the decision between clipping or EVT up to randomization (is there clinical equipoise)?^b^ (%)															
Yes	1 (20)	0 (0)	0 (0)	2 (40)	0 (0)	0 (0)	0 (0)	0 (0)	2 (40)	0 (0)	1 (20)	2 (40)	0 (0)	0 (0)	0 (0)
No	4 (80)	5 (100)	5 (100)	3 (60)	1 (20)	0 (0)	4 (80)	3 (60)	3 (60)	5 (100)	4 (80)	3 (60)	5 (100)	5 (100)	5 (100)
Not applicable (no primary treatment)	0 (0)	0 (0)	0 (0)	0 (0)	4 (80)	5 (100)	1 (20)	2 (40)	0 (0)	0 (0)	0 (0)	0 (0)	0 (0)	0 (0)	0 (0)
Do you agree with the following statement “There was no doubt in our decision for the primary treatment modality (endovascular or clipping)”? (%)															
Strongly agree	3 (60)	3 (60)	4 (80)	2 (40)	0 (0)	0 (0)	4 (80)	2 (40)	4 (80)	5 (100)	3 (60)	3 (60)	4 (80)	5 (100)	2 (40)
Agree	1 (20)	2 (40)	1 (20)	1 (20)	1 (20)	0 (0)	0 (0)	0 (0)	1 (20)	0 (0)	1 (20)	1 (20)	1 (20)	0 (0)	2 (40)
Disagree	1 (20)	0 (0)	0 (0)	1 (20)	0 (0)	0 (0)	0 (0)	0 (0)	0 (0)	0 (0)	0 (0)	1 (20)	0 (0)	0 (0)	1 (20)
Strongly disagree	0 (0)	0 (0)	0 (0)	1 (20)	0 (0)	0 (0)	0 (0)	0 (0)	0 (0)	0 (0)	1 (20)	0 (0)	0 (0)	0 (0)	0 (0)
Not applicable (no primary treatment)	0 (0)	0 (0)	0 (0)	0 (0)	4 (80)	5 (100)	1 (20)	3 (60)	0 (0)	0 (0)	0 (0)	0 (0)	0 (0)	0 (0)	0 (0)

Abbreviations: ELD, external lumbar drain; EVD, external ventricular drain; EVT, endovascular treatment; MDT, multidisciplinary team.

a Up to three reasons were allowed per case.

b Is there, in the opinion of the MDT, clinical equipoise in this case e.g. does the MDT expect similar outcomes between EVT or clipping in this case.

Supplementary, for each case, the MDTs were asked whether they would perform CSF drainage, by what modality (ventricular or lumbar), and at what point relative to the primary treatment (before, concomitant, or after).

### 2.4 Statistical analysis

Data were primarily described using percentages, medians (with interquartile ranges [IQR], standardized mean differences (SMD) and p-values. Fleiss' κ statistic and its 95% CI were used to quantify between-center agreement for aneurysm treatment modality (Landis and Koch, 1977). We explored the association of median MDT experience in years and aneurysm treatment decisions using Spearman's rank correlation coefficients (Schober et al., 2018). Thus, for each MDT the median years of experience across all participating medical specialists was calculated. Statistical analyses were performed using R version 4.4.0. Missing data were not imputed and reported where applicable.

### 2.5 Ethics and informed consent

The study used data from the SPARTA cohort (ClinicalTrials.gov registration number: NCT05851989), which was approved by the Medical Ethics Committee (METC LDD). Written informed consent was obtained from all participants or their legal representatives in accordance with the SPARTA protocol (Hamming et al., 2024). If patients died before the informed consent procedure could be discussed and there was no legally appropriate shared decision making, inclusion proceeded under deferred consent, unless a documented objection to participation in research had been documented in the medical file (Hamming et al., 2024; Koopman et al., 2022; van Wijk et al., 2020). For patients included under deferred consent, legal representatives or next of kin, if available, were subsequently approached to provide permission for the use of the patient's data in this study.

## 3 Results

### 3.1 Multidisciplinary team characteristics

A total of 16 medical specialists (4 [interventional] neurologists, 8 neurosurgeons (n = 2 hybrids as well) and 4 [interventional] radiologists, Supplemental Table 2) participated in the MDT meetings across five centers. 14 participants (88%) were male. The median age was 47 years (range 35 – 65) and the median time since completion of residency was 13.5 years (range 1– 33). MDT median years of post-residency experience showed no statistically significant correlation with the proportion of cases treated endovascularly (Spearman ρ: 0.63, p = 0.25) or clipping (Spearman ρ: −0.31, p = 0.61). Spearman's coefficient 95% CI calculation through bootstrap resampling was not feasible due to sample size limitations (5 MDTs).

### 3.2 Aneurysm treatment decisions

**Fig. 1:**
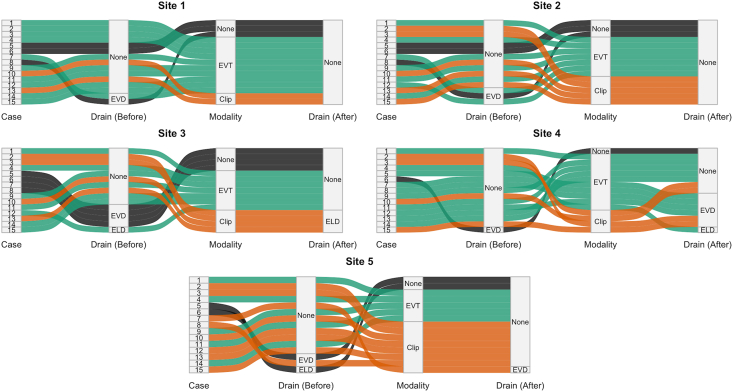
Sankey diagram of treatment strategy for all 15 case vignettes and centers. *Abbreviations:* Clip, microsurgical clipping; CSF, cerebrospinal fluid; ELD, external lumbar drainage; EVD, External ventricular drainage; EVT, endovascular treatment; None, no primary treatment; PT, primary treatment; TLD, treatment limiting decision-making.

The MDTs reached between-center consensus on whether there was an indication to perform aneurysm treatment in 11 of 15 case vignettes (73%, Table 2, and Fig. 1). In one case (6) there was consensus to not perform aneurysm treatment. In the three remaining cases (5, 7, and 8), there was between-center disagreement on the decision to perform aneurysm treatment (20%, 80%, and 40%, respectively). The most frequently cited reasons for not performing aneurysm treatment were poor neurological condition (13/13 total treatment withholding decisions, 100%) and advanced age (7/13, 54%, Table 2).

### 3.3 Aneurysm treatment modality

In the 11 case vignettes for which all centers proposed aneurysm treatment, at least four out of five centers agreed on the aneurysm treatment modality in 10 cases (91%). Consensus across all five centers was reached in 4 out of 11 cases (36%).

**Fig. 2:**
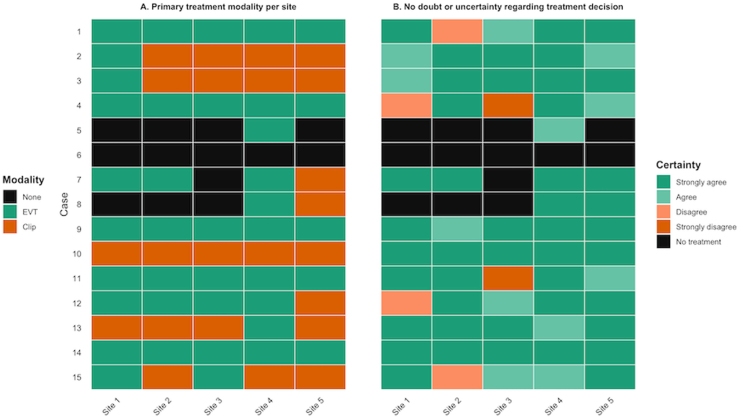
Heatmap of primary treatment modality choice and certainty for all 15 case vignettes and all centers. *Abbreviations:* Clip, microsurgical clipping; EVT, endovascular treatment (such as bare coiling, stent-assisted coiling and balloon-assisted coiling); None, no primary treatment (initial conservative management or treatment limiting decision-making).

Across all proposed treatments, a total of 62 treatment strategies (75 total strategies minus 13 treatment withholding decisions) were formulated. Bare (simple) coiling was the most frequently proposed aneurysm treatment modality (37/62, 60%, Table 2 & Supplemental Fig. 2), followed by microsurgical clipping (22/62, 35%). Stent-assisted and balloon-assisted coiling were rarely proposed (1/62, 2% and 2/62, 3%, respectively), and none of the centers recommended treatment with an intrasaccular device. The proportion of opting for clipping as treatment modality varied across centers, ranging from 2 out of 12 (17%) of proposed aneurysm treatments within that center to 8 out of 13 (62%) of proposed aneurysm treatments within that center (Fig. 2). Between-center agreement was moderate for aneurysm treatment modality (Fleiss' κ = 0.49, 95%CI 0.31-0.68, Fig. 4), but increased when adjusted for category prevalence (Gwet's AC1 = 0.62, 95% CI 0.46-0.78, raw agreement: 68%). Agreement significantly decreased when decisions regarding CSF drainage modality (AC1 = 0.39, 95% CI 0.2-0.58, raw agreement: 44%) and drainage timing (AC1 = 0.37, 95% CI 0.18-0.57, raw agreement 41%) were included in the treatment strategy.

Across the 25 MDT aneurysm treatment modalities per aneurysm location (five centers each assessing five cases per aneurysm location), ACOM and PCOM aneurysms were predominantly treated endovascularly, with bare coiling recommended in 14/25 (56%) ACOM cases and 19/25 (76%) PCOM cases. Contrarily, MCA aneurysms were mostly treated with clipping (20/25 MCA cases, 80%, Supplemental Fig. 1).

**Fig. 3:**
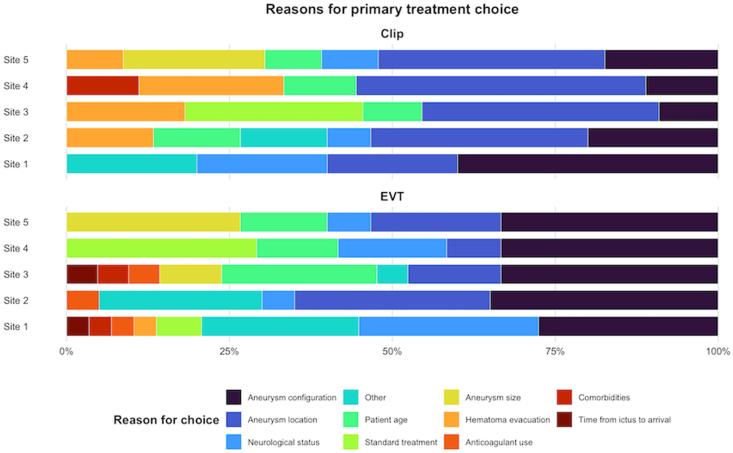
Reasons for primary treatment modality choice (EVT versus clipping) *Abbreviations:* Clip, microsurgical clipping; EVT, endovascular treatment (such as bare coiling, stent-assisted coiling and balloon-assisted coiling).

Centers were asked to provide up to three reasons for their choice of aneurysm treatment modality. In total, 185 reasons for aneurysm treatment modality were given. Out of the 37 aneurysm treatment proposals where the MDT opted for bare coiling, 103 total reasons were provided. The most common reasons were aneurysm configuration (33/103, 32%), patient's neurological condition in (14/103, 14%), and aneurysm location in (13/103, 13%). Out of the 22 aneurysms treatment proposals where the MDT opted for clipping, 60 total reasons where provided. The main reasons were aneurysm location in (21/60, 35%), presence of a concomitant intracranial hemorrhage in (11/60 18%), and aneurysm configuration in (10/60, 17%, Table 2, and Fig. 3).

**Fig. 4:**
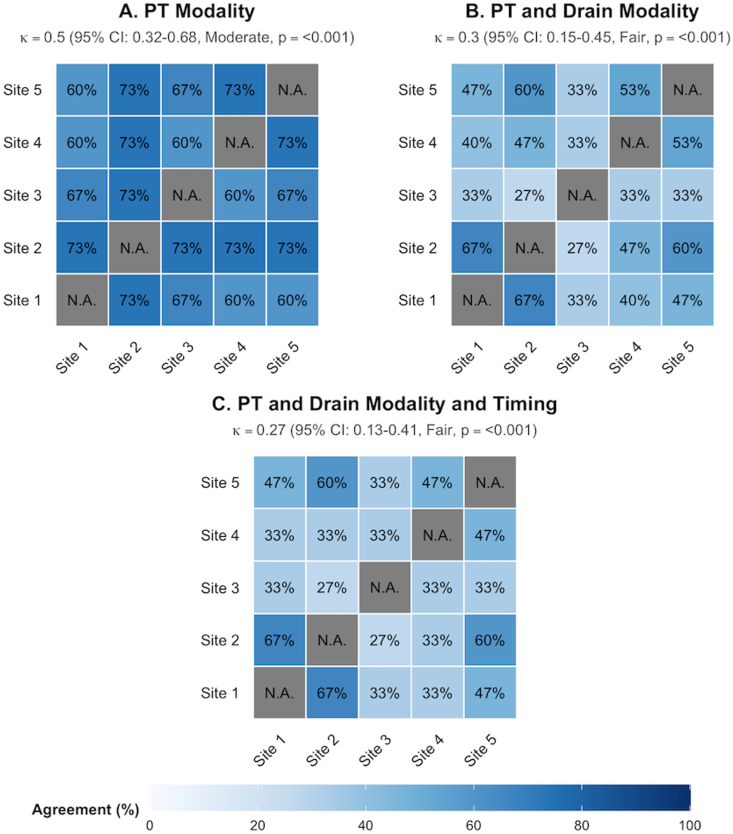
Pairwise between-center agreement across all cases Abbreviations: CI, confidence interval; κ, Fleiss' kappa; N.A., not applicable; p, probability value; PT, primary treatment.

### 3.4 Clinical equipoise in treatment decisions

In 5 cases, at least one of the MDTs indicated a willingness to randomize the choice of clipping versus endovascular treatment, reflecting the presence of clinical equipoise (Table 2). However, the presence of clinical equipoise was reported in only 8 times out of 62 treatment decisions, despite between-center variations in treatment choice. Moreover, most MDTs reported high certainty in their decisions. In 44 of 62 (71%) treatment proposals, MDTs strongly agreed they had no doubt about their chosen aneurysm treatment modality (Fig. 2). In contrast, only 4 of 62 (6%) disagreed and 2 of 62 (3%) strongly disagreed that there was no doubt in their choice, indicating that substantial uncertainty was uncommon.

### 3.5 CSF drainage practices

The proportion of the application of CSF drainage versus no CSF drainage differed considerably between centers, ranging from 2/15 (13%) to 9/15 (60%). Practices also varied in choice of drainage modality and timing relative to aneurysm treatment (Fig. 1). One center consistently favored lumbar drainage after microsurgical clipping (Fig. 1). Full agreement on drainage strategy (modality and timing) was reached in six cases (40%).

## 4 Discussion

In this multicenter case vignette study, there is between-center variation in the proposed management strategies for identical aSAH cases. Most centers agreed on the indication of aneurysm treatment. Contrarily, there was between-center heterogeneity in choice of treatment modality, the level of certainty in the proposed treatment strategies, and the application of CSF drainage.

Unlike prior studies on inter-center variability, our vignette-based design isolates decision-making from case-mix by presenting identical cases, allowing direct comparison of treatment preferences. Our findings suggest the persistence of practice variation more than two decades after the ISAT trial. Because both treatment modalities carry different risk and complication profiles, unwarranted variation may contribute to differences in clinical outcomes, although determining whether such variation is unwarranted requires evaluation of center-level treatment strategies against patient outcomes (Birkmeyer et al., 2013/09; Atsma et al., 2020). This heterogeneity underscores the challenges of translating evidence in complex neurovascular care, where patient heterogeneity, institutional norms, and procedural experience may outweigh generalized trial findings in shaping practice patterns.

Notably, we observed limited uncertainty in treatment choices and willingness of centers to randomize aneurysm treatment modality, indicative of a limited perception of clinical equipoise, even in cases with high between-center disagreement (Alexander, 2022/03). Such a paradox has implications for the feasibility of future randomized trials in neurovascular care, where embedded institutional preferences may limit recruitment (Singh et al., 2024). In this context, prospective observational approaches offer a promising alternative, especially when using center-level preferences for comparative effectiveness research (Cnossen et al., 2018; Baiocchi et al., 2014).

We found clear center-level preferences in treatment strategies for aSAH between the participating Dutch centers, independent of case-mix. These preferences may reflect differences in institutional expertise and experiences, entrenched practice patterns or alternative interpretations of the current literature (Lagares et al., 2015; de Winkel et al., 2021; Birkmeyer et al., 2013/09). Because reimbursement for neurovascular procedures in the Netherlands is regulated centrally and uniformly across hospitals, potential financial incentives are unlikely to account for differences in treatment strategies. Center-level treatment preferences may serve as a natural experiment for comparative effectiveness research (Cnossen et al., 2018; van Essen et al., 2022/07; Baiocchi et al., 2014; Vreeburg et al., 2024). For instrumental variable analysis to be valid, certain assumptions must be met. One of which is that the instrument (in this case, the treating center) must be correlated with the exposure, here the treatment modality (clipping or endovascular treatment) (Cnossen et al., 2018; Baiocchi et al., 2014; Widding-Havneraas and Zachrisson, 2022/09). Our findings support the plausibility of these assumptions, demonstrating treatment preferences across centers that were not attributable to case-mix, as all centers evaluated identical case vignettes. Leveraging these differences could strengthen causal inference claims in future outcome studies, and potentially reduce the necessity for randomized clinical trials, where recruitment may be impaired by profound institutional treatment preferences and a low perception of clinical equipoise (Hamming et al., 2024).

Supplementary, between-center variation in primary treatment modality was accompanied by differences in CSF drainage strategies, including modality and timing. Some consistently favored lumbar drainage after clipping, while other centers preferred placing ventricular drains during primary treatment. These differences are clinically relevant, given the competing risks associated with CSF drainage, mainly infections. CSF drainage modality and timing are associated with differing complication profiles and may influence patient outcome (Wolf et al., 2023; Chao et al., 2024; An et al., 2025). Notably, the EARLYDRAIN randomized trial demonstrated clinical benefit of early, protocolized lumbar drainage by reducing secondary infarction and unfavorable outcome. We demonstrated that the adoption of protocolized lumbar drainage similarly varies across centers, and, given its association with outcome, might therefore violate the exclusion restriction assumption for instrumental variable analysis in future comparative effectiveness studies on primary treatment modality of aneurysm repair (Wolf et al., 2023).

There are several limitations that merit consideration. First, although systematically presenting case vignettes allowed us to isolate treatment preferences independent of case-mix, they cannot fully capture the complexity of certain cases. Second, the study was limited to five Dutch tertiary referral centers for aSAH, which may limit generalizability to other countries and regions in the world. However, the presence of variation between centers within a single country and even within regions might indicate that global variation is likely to be greater than less (de Winkel et al., 2021; van Essen et al., 2022/07; Birkmeyer et al., 2013/09; Vreeburg et al., 2025). Third, while the number of case vignettes was modest, they represented diverse clinical phenotypes of aSAH, making it unlikely that larger numbers would have altered the main conclusions (Hillen et al., 2025/07). Fourth, the study selected real-world cases from the SPARTA observational cohort, which meant that not all imaging was performed for every patient. In certain case vignettes, a digital subtraction angiography (DSA) would be missing while certain participating centers indicated that they required a DSA to recreate real-world clinical decision-making. Fifth, MDT members may have been aware of the patient's eventual living status (death or alive) when radiologic images were displayed in the medical file. Since strict de-identification was not feasible for all materials, this may have influenced treatment decisions and represents an inherent limitation of the vignette-based design (Plaum et al., 2024). Although, this information was unlikely to have influenced treatment choices because outcomes may have occurred long after the initial hemorrhage and potentially unrelated to the aSAH. Sixth, we did not differentiate between initial conservative management and treatment-limiting decisions within the “no primary treatment” category, since such distinctions are often not yet clearly defined at initial presentation, prognostic uncertainty remains high and the clinical condition may change over time. While this approach reflects real-world early decision-making, it limited the ability to distinguish between fundamentally different clinical and ethical considerations underlying these choices. Finally, aSAH management is not limited to aneurysm occlusion strategy and CSF diversion, but also extends beyond these decision, including neurocritical care such as ICP control, blood pressure management, and vasospasm monitoring. Therefore, our findings reflect variability in early treatment strategies rather than the full spectrum of aSAH management.

## 5 Conclusion

There is between-center variation in treatment strategies for ruptured intracranial aneurysms across Dutch tertiary referral centers for aSAH in identical case vignettes. These case-mix independent, center-level preferences may be leveraged for comparative effectiveness research.

## Reproduced and Re-created material

All information and materials in the manuscript are original.

## Previous presentations

None.

## Previous publications

None.

## Participating MDT specialists and their corresponding affiliations

**AMC**.

Dr. *R. van* de Berg, Interventional-radiologist, Department of Radiology.

Dr. B. Coert, Neurosurgeon, Department of Neurosurgery.

Drs. I. Kommers, Neurosurgeon, Department of Neurosurgery.

Dr. C. Puylaert, Interventional-radiologist, Department of Radiology.

Dr. M. Lequin, Neurosurgeon, Department of Neurosurgery.

**HMC**.

Dr. J. van Dijck, Neurosurgeon, Department of Neurosurgery.

Dr. K. Jellema, Neurologist, Department of Neurology.

Dr. *I. van* de Wijngaard, Interventional-neurologist, Department of Radiology and Neurology.

**RUMC**.

Dr. J. de Vries, Neurosurgeon, Department of Neurosurgery.

Prof. dr. J. Boogaarts, Neurosurgeon, Department of Neurosurgery.

**MUMC**.

Dr. R. Haeren, Neurosurgeon, Department of Neurosurgery.

Drs. B. Wagemans, Interventional-radiologist, Department of Radiology.

Dr. I. de Ridder, Neurologist, Department of Neurology.

**UMC Utrecht**.

Dr. T. van Doormaal, Neurosurgeon, Department of Neurology and Neurosurgery.

Dr. M.D.I. Vergouwen, Neurologist, Department of Neurology and Neurosurgery.

Prof. dr. I.C. van der Schaaf, Interventional-radiologist, Department of Radiology.

## Disclosure of funding

The SPARTA study was sponsored by the Sint Jacobus Stichting, a non-profit organisation. The sponsor has no role in the design of the study and the collection, analysis and interpretation of data.

## Conflict of interest

None.

## Figures and Tables

**figs1:**