# Supplementary material for: Treatment variation in acute management of patients with aneurysmal subarachnoid hemorrhage: a multicenter case vignette study
Source: Brain Spine. 2026 May 5;6:106071. doi: 10.1016/j.bas.2026.106071 (PMC13196106; doi:10.1016/j.bas.2026.106071)
Supplement: Multimedia component 1 [file mmc1.docx]

**Case Report Form (CRF)**

***General questions (to be filled in by researcher present at the multidisciplinary team meetings [MDT])***

Question 1. In which institution are the cases currently discussed?

|  |
| --- |

Question 2. Which specialists are currently present during the MDT meetings?

| **Specialism** | **Number of specialists present** | **Years of experience** | **Age(s)** | **Sex(es)** |
| --- | --- | --- | --- | --- |
| Neurosurgeon |  |  |  |  |
| Neurologist |  |  |  |  |
| Interventional-neuroradiologist |  |  |  |  |
| Interventional-neurologist |  |  |  |  |

**Case 1 – 2 – 3 – 4 – 5 – 6 – 7 – 8 – 9 – 10 – 11 – 12 – 13 – 14 – 15**

**1. Do you see an indication for cerebrospinal fluid (CSF) drainage?**

- 1. Yes, ventricular
  2. Yes, lumbar
  3. No

**2. If CSF drainage was chosen in Question 1, would you perform the drainage before or after aneurysm treatment?**

a. Before aneurysm treatment

b. After aneurysm treatment

c. No preference

**3. Would you primarily treat (aneurysm repair) this patient at this time?**

a. Endovascular intervention / clipping

b. None ([initially] conservative or treatment-limiting decision making)

c. Other (excluding CSF drainage), specify:…

|  |
| --- |

**4. If no primary treatment was chosen in Question 3: what are the primary reasons for your choice in Question 3? (Multiple answers possible, up to 3 reasons per case)**

a. Neurological status (GCS, Hunt & Hess, WFNS)

b. Patient age

c. Presence or absence of comorbidities

d. Presence or absence of multiple aneurysms

e. Time between ictus (onset) and arrival at the treatment center

f. Use of anticoagulants

g. Aneurysm configuration

h. Aneurysm size

i. Aneurysm location

j. Other, namely:…

|  |
| --- |

**5. If primary treatment was chosen in Question 3, which treatment would be most suitable in your opinion?**

a. Clip reconstruction

b. “Bare” coiling

c. Balloon-assisted coiling

d. Stent-assisted coiling

e. Flow-diverter

f. Other, specify:…

|  |
| --- |

**6. What are the 3 primary reasons for your choice in Question 5? (Multiple answers possible, up to 3 reasons per case)**

a. Neurological status (GCS, Hunt & Hess, WFNS)

b. Standard hospital protocol/practice

c. Patient age

d. Presence or absence of comorbidities

e. Presence or absence of multiple aneurysms

f. Time between ictus (onset) and arrival at the treatment center

g. Use of anticoagulants

h. Aneurysm configuration

i. Aneurysm size

j. Aneurysm location

k. Other, namely:…

|  |
| --- |

**7. Would you be willing to leave the choice between clip reconstruction or endovascular treatment to randomization? (e.g., is there "clinical equipoise" in your opinion?)**

a. Yes

b. No

**8. Do you agree with the following statement: “There was no doubt or hesitation in our decision for clipping or endovascular treatment”?**

a. Strongly agree

b. Agree

c. Disagree

d. Strongly disagree
