# Supplementary material for: Treatment variation in acute management of patients with aneurysmal subarachnoid hemorrhage: a multicenter case vignette study
Source: Brain Spine. 2026 May 5;6:106071. doi: 10.1016/j.bas.2026.106071 (PMC13196106; doi:10.1016/j.bas.2026.106071)
Supplement: Multimedia component 2 [file mmc2.docx]

**Supplemental Table 1 | Radiological images of each case presented to the MDTs**

| 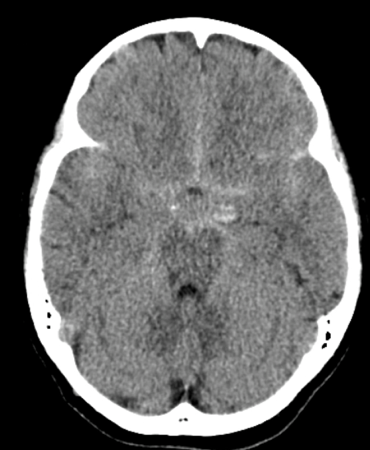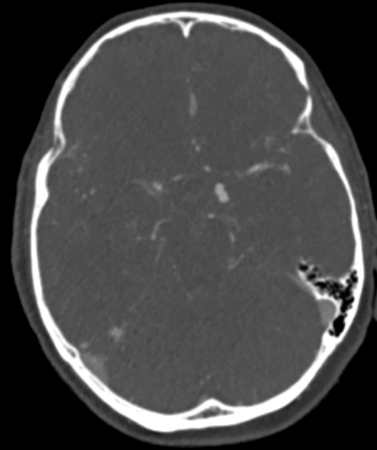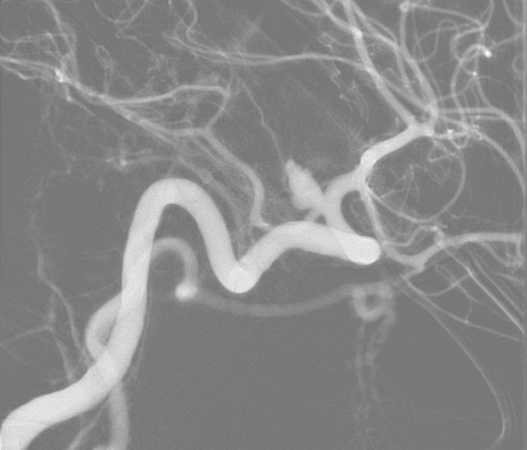  **Case 1** |
| --- |
| 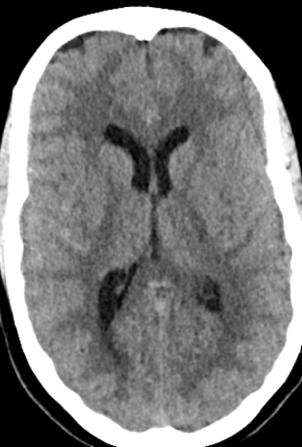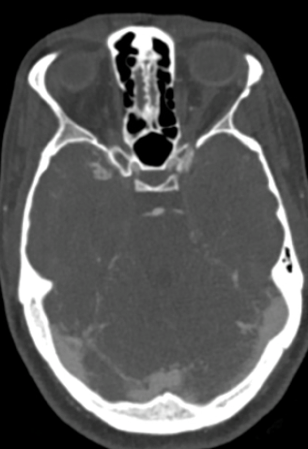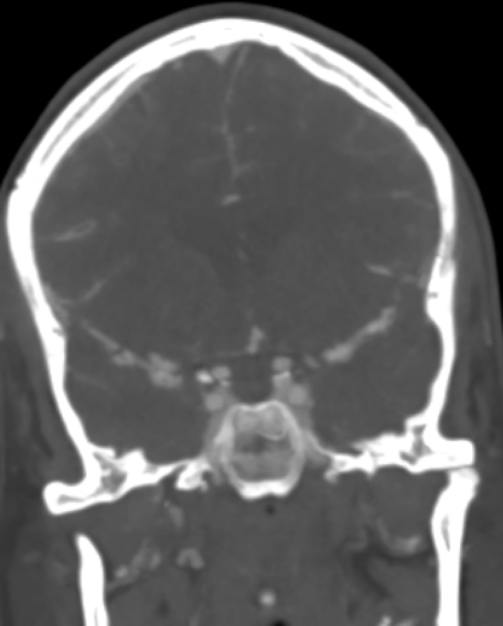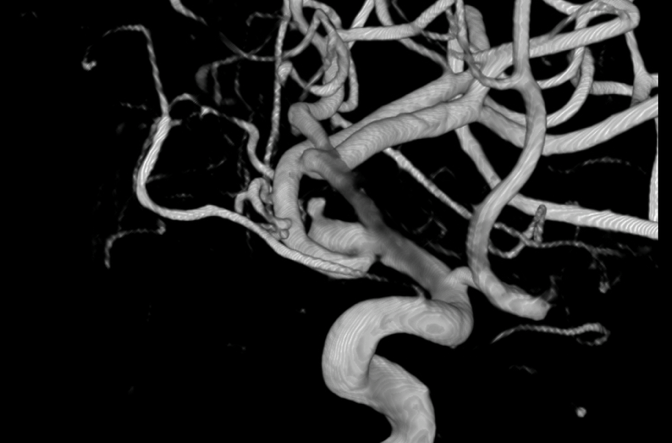  **Case 2** |
| 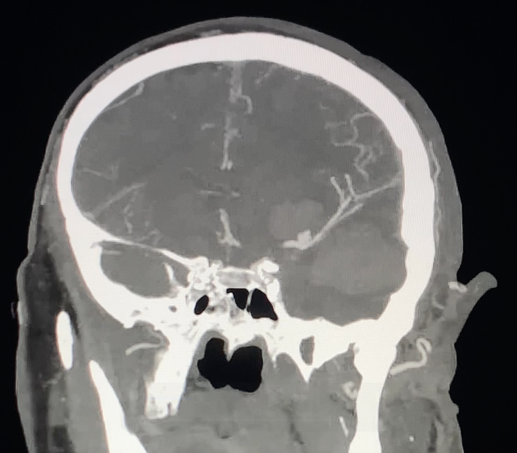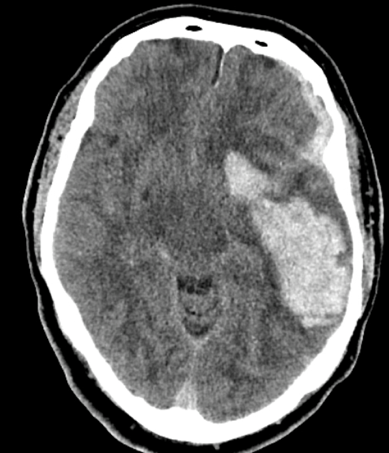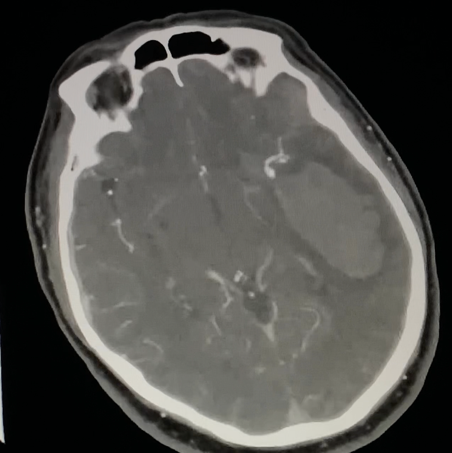  **Case 3^a^** |
| 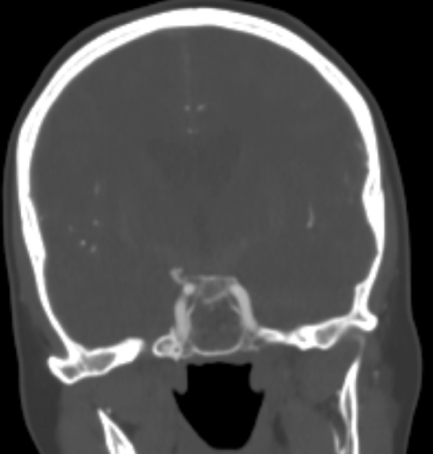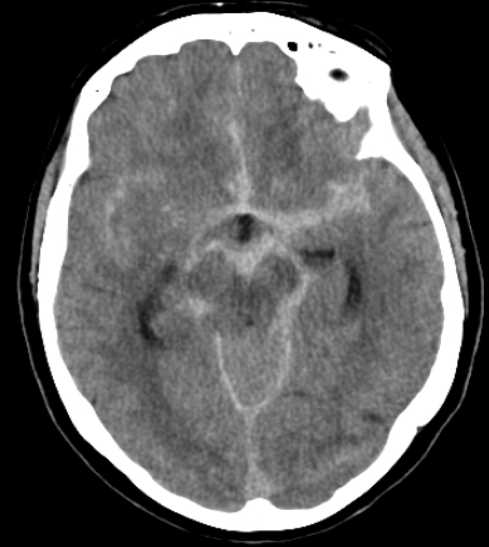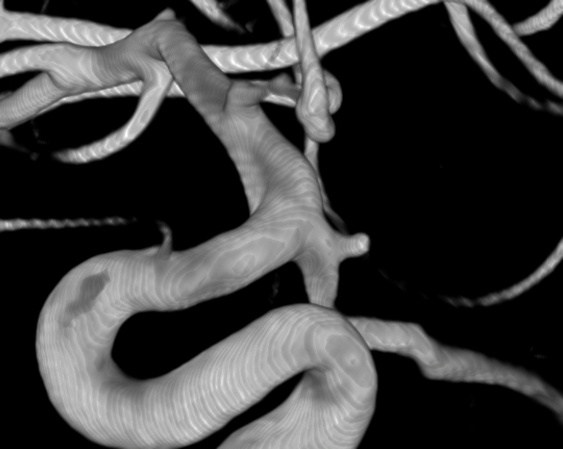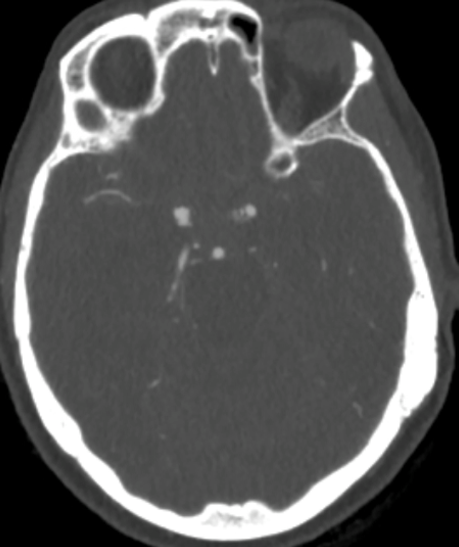  **Case 4** |
| 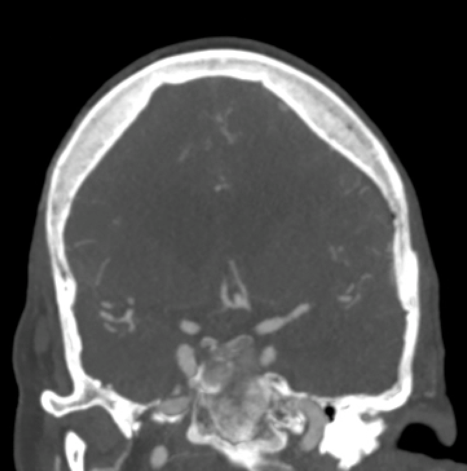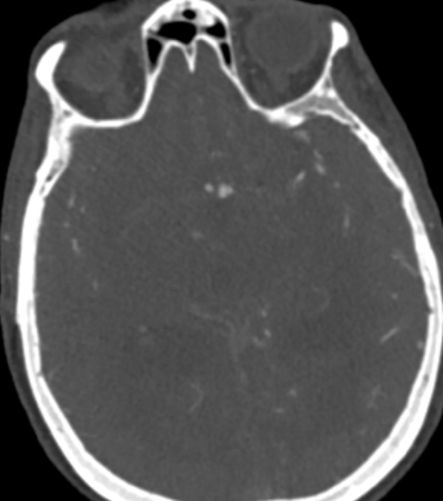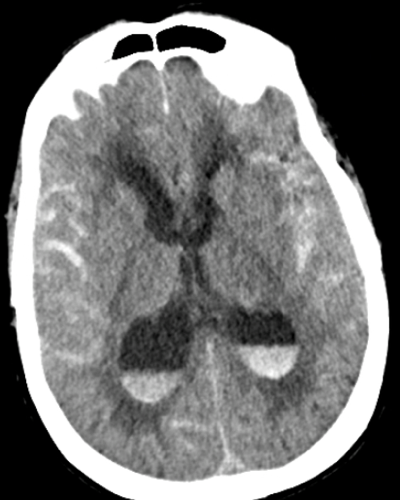  **Case 5^a^** |
| 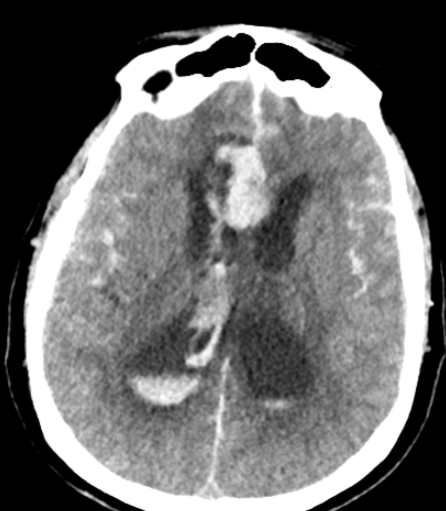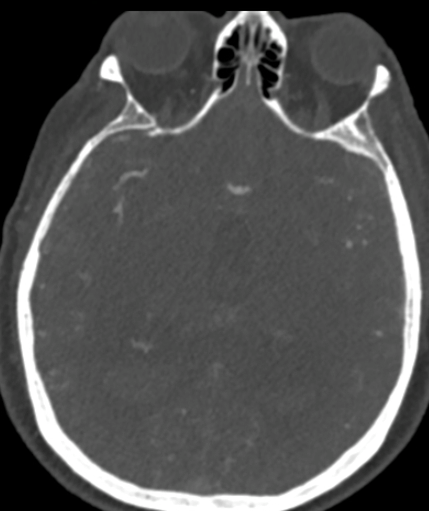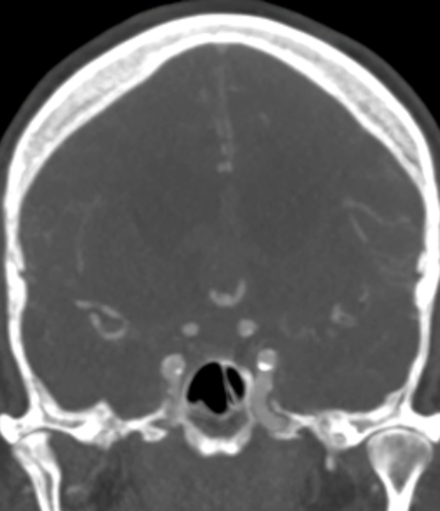  **Case 6^a^** |
| 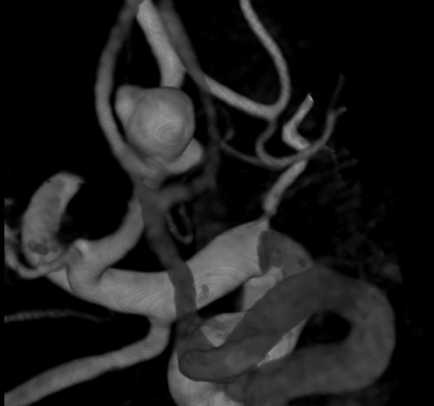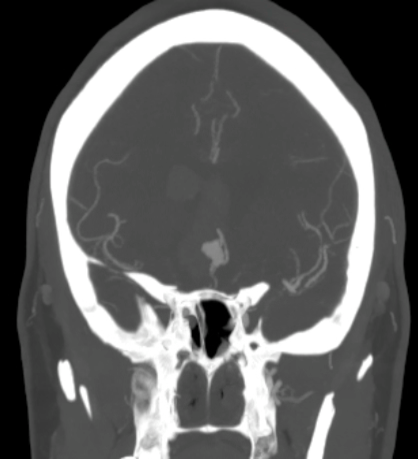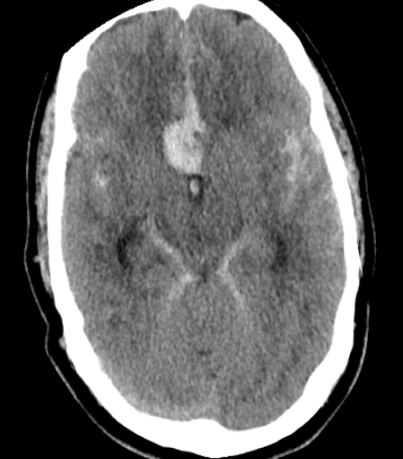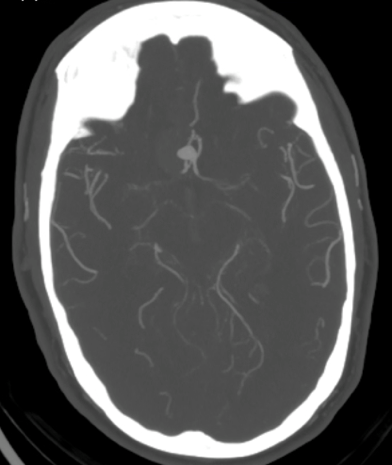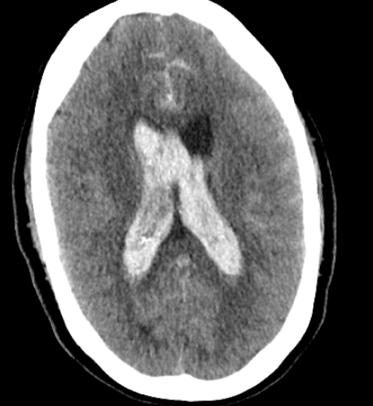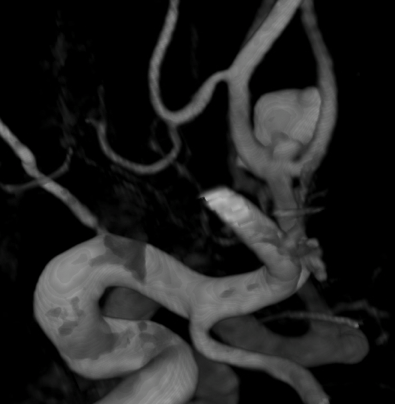  **Case 7** |
| 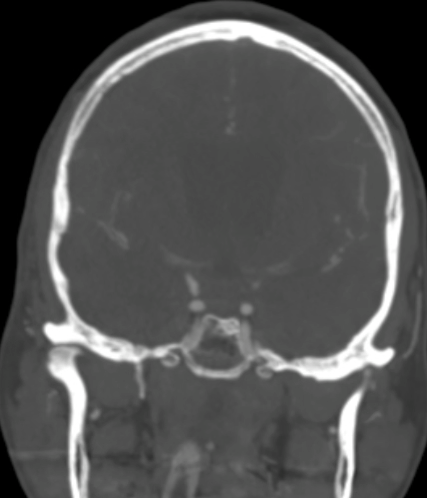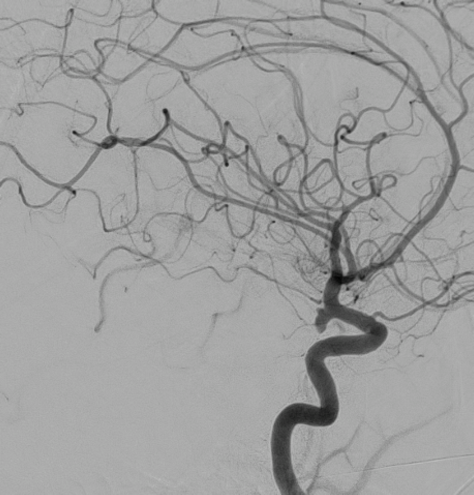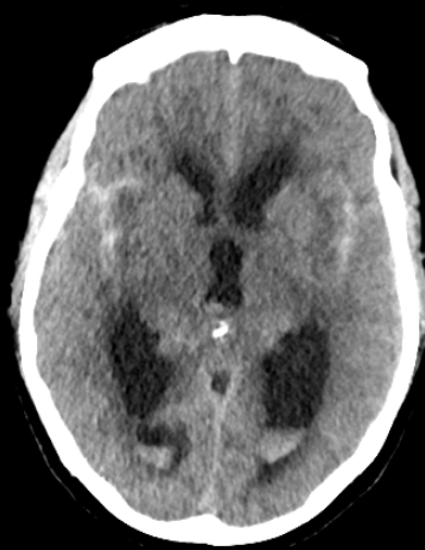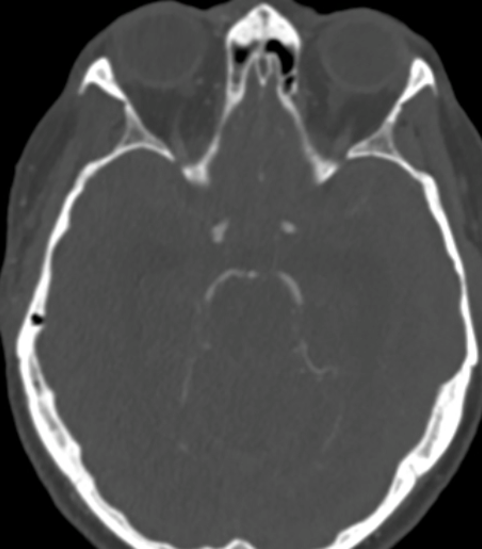  **Case 8** |
| 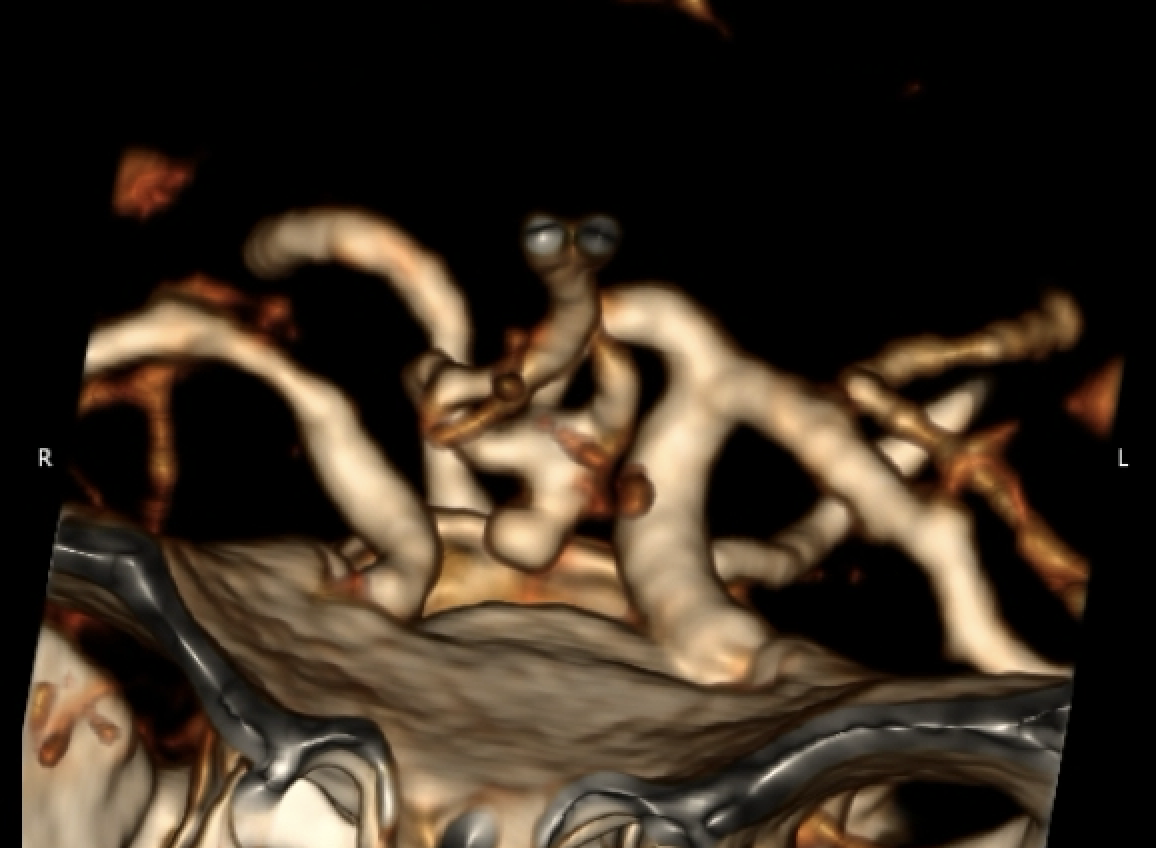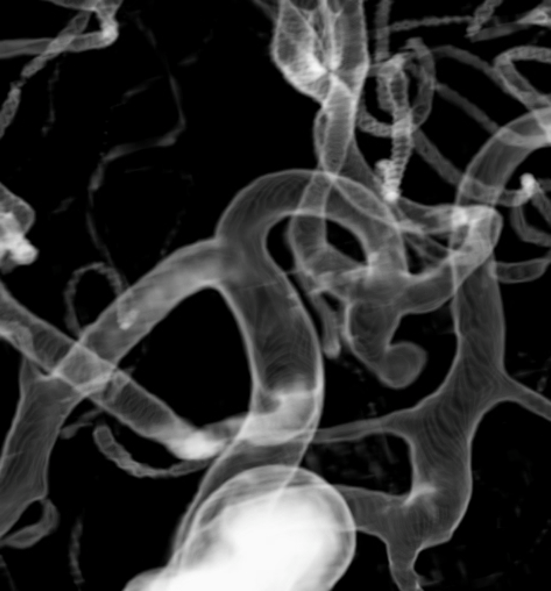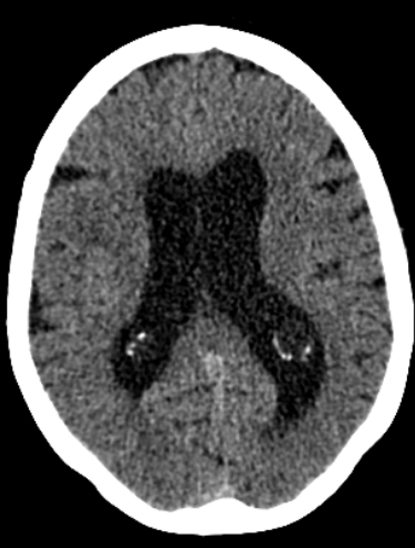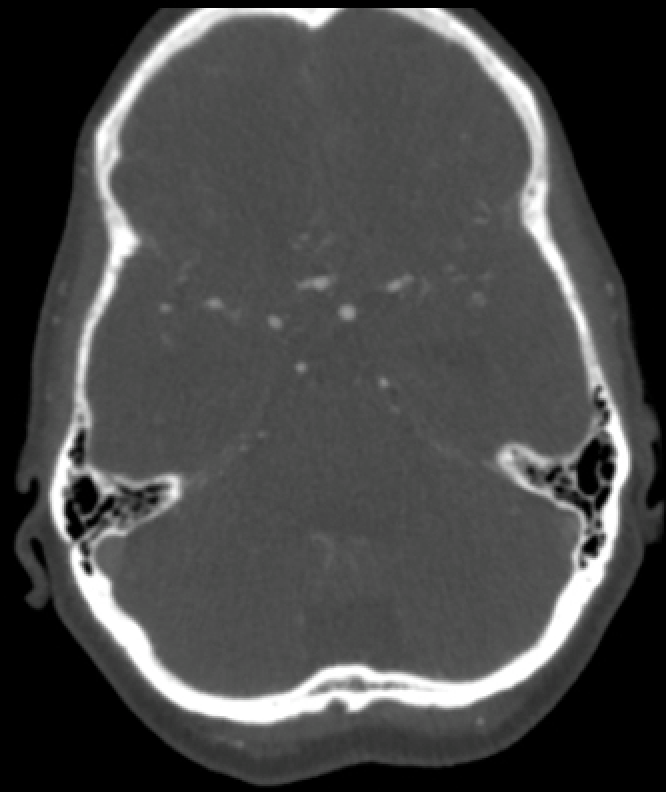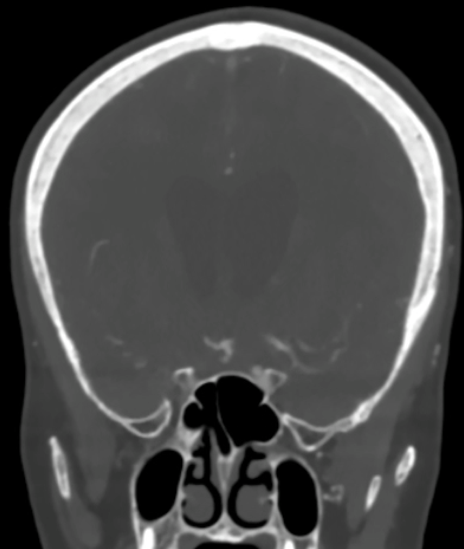  **Case 9** |
| 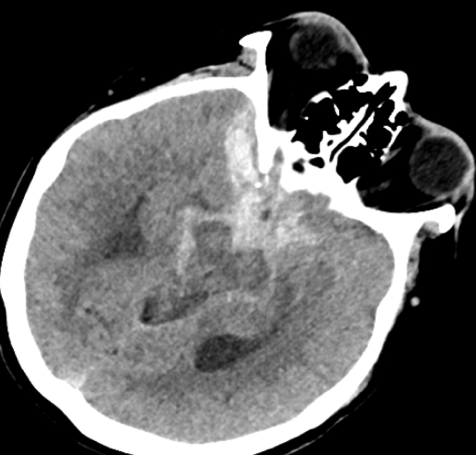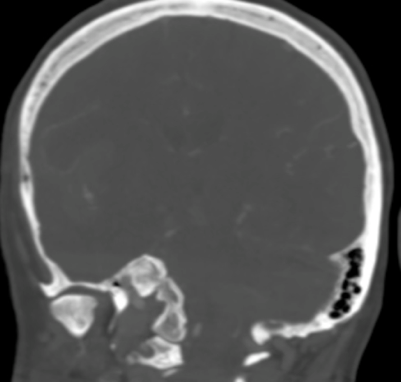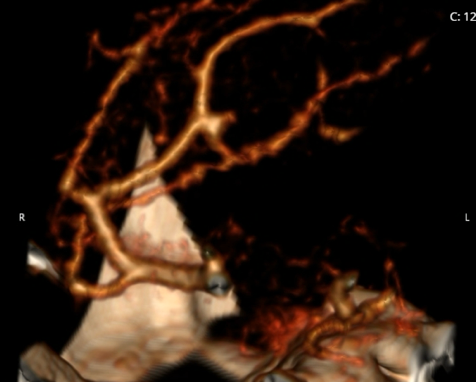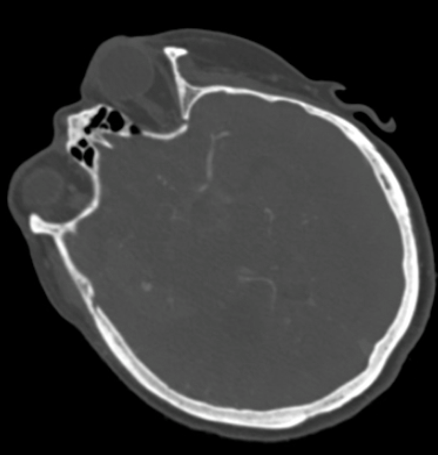  **Case 10** |
| 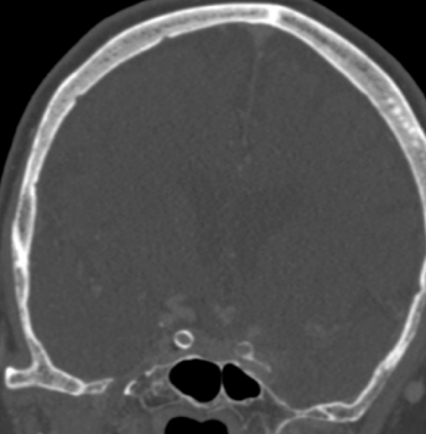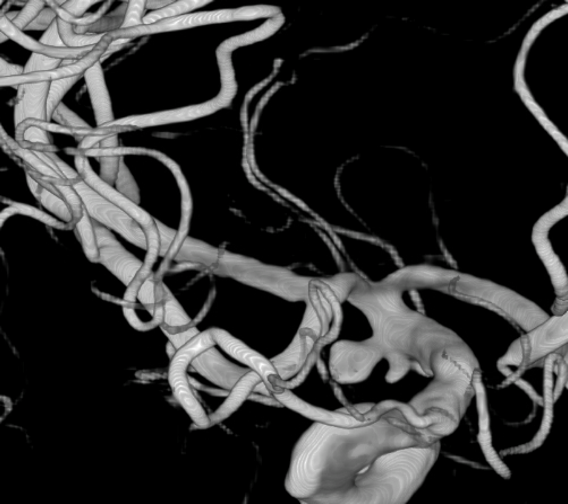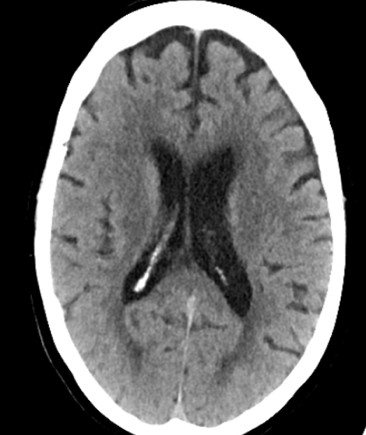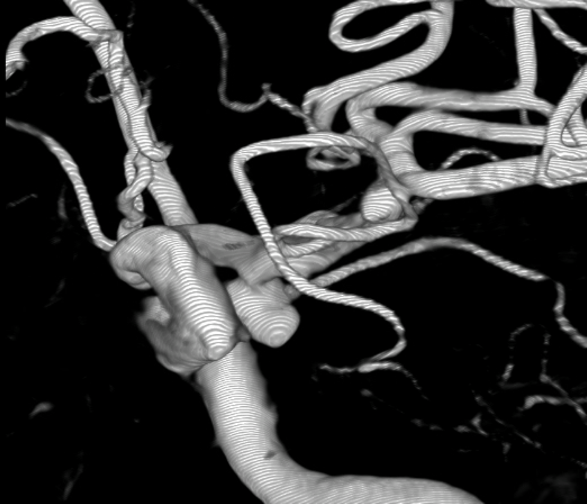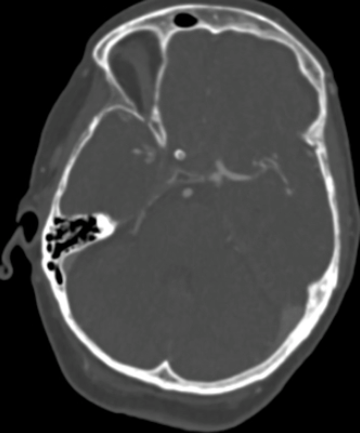  **Case 11** |
| 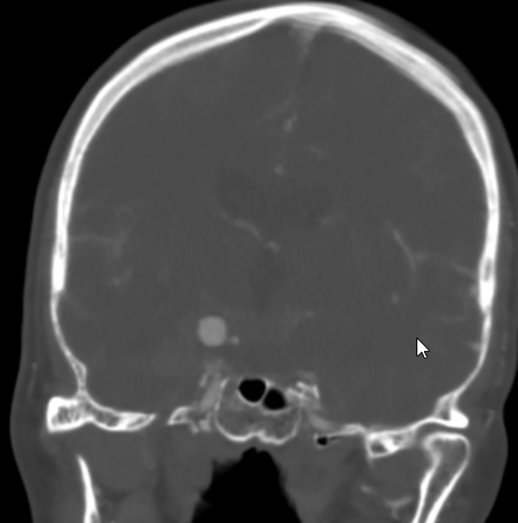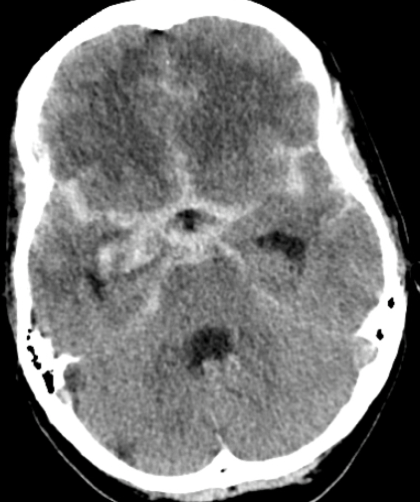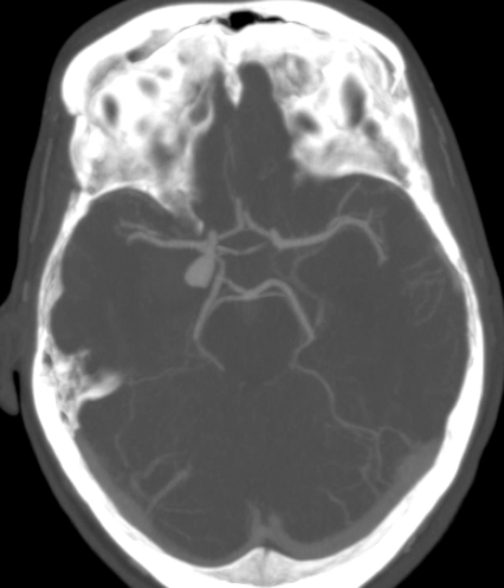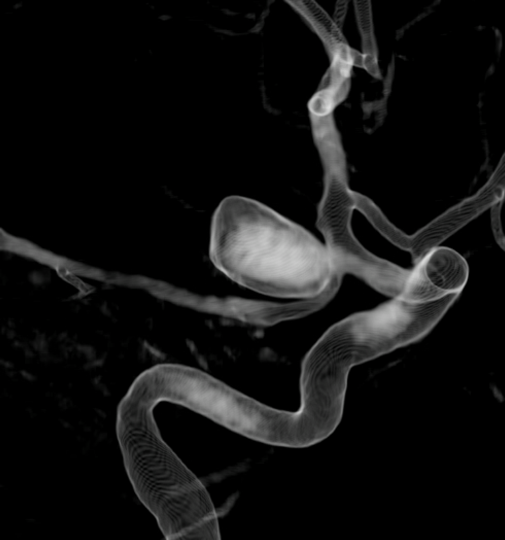  **Case 12** |
| 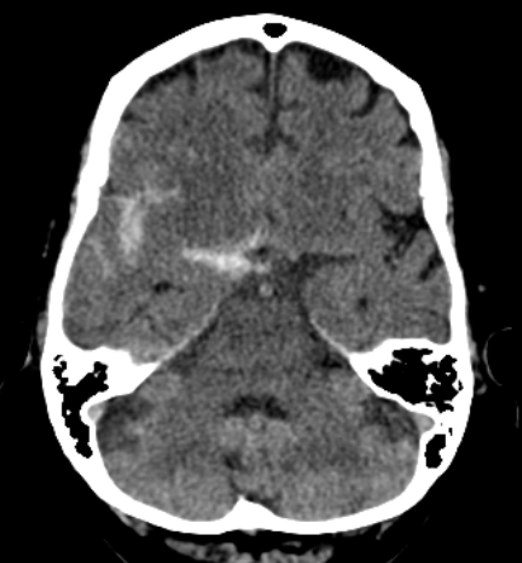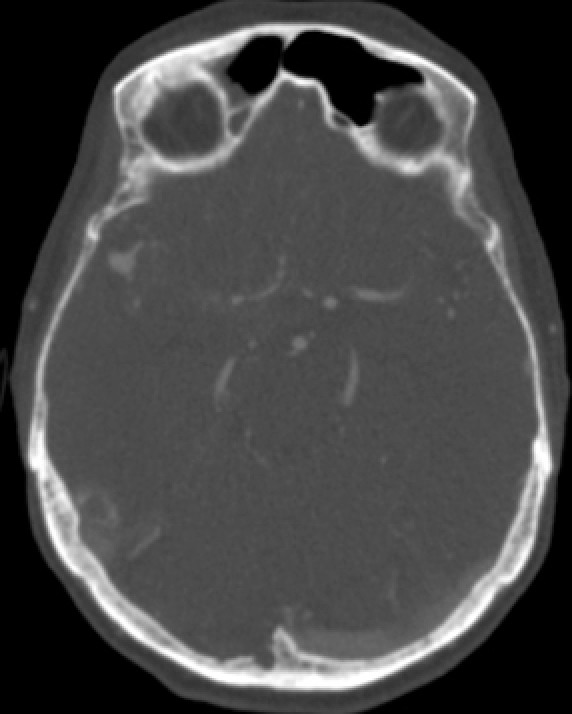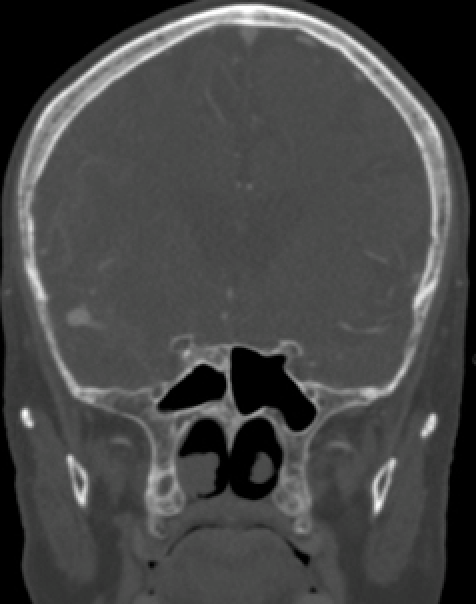  **Case 13^a^** |
| 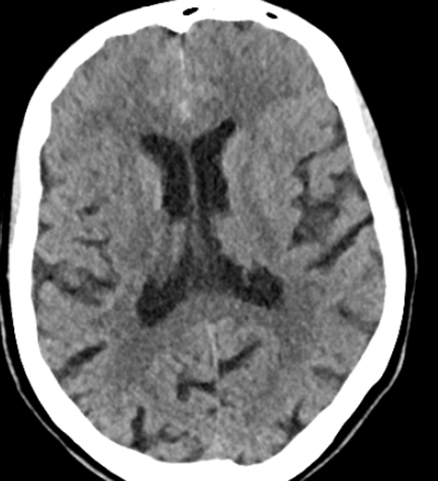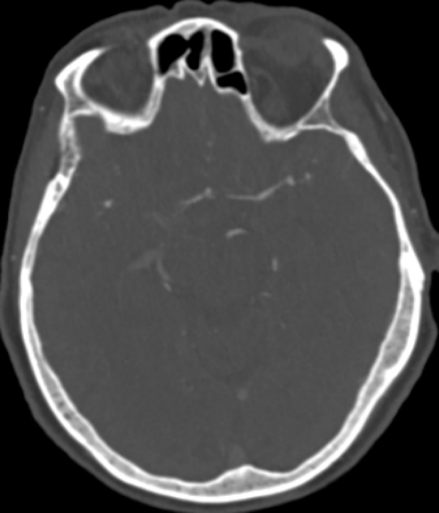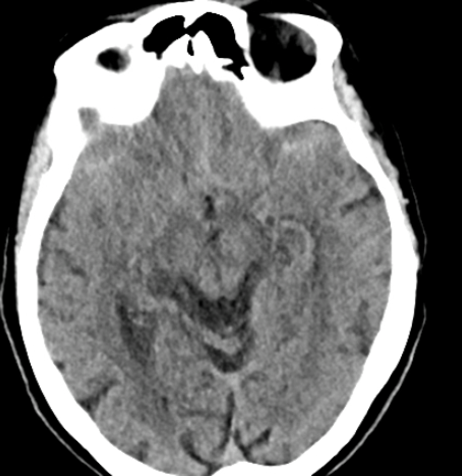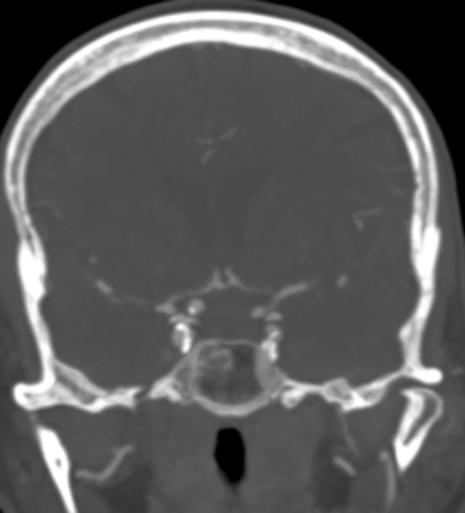  **Case 14^a^** |
| 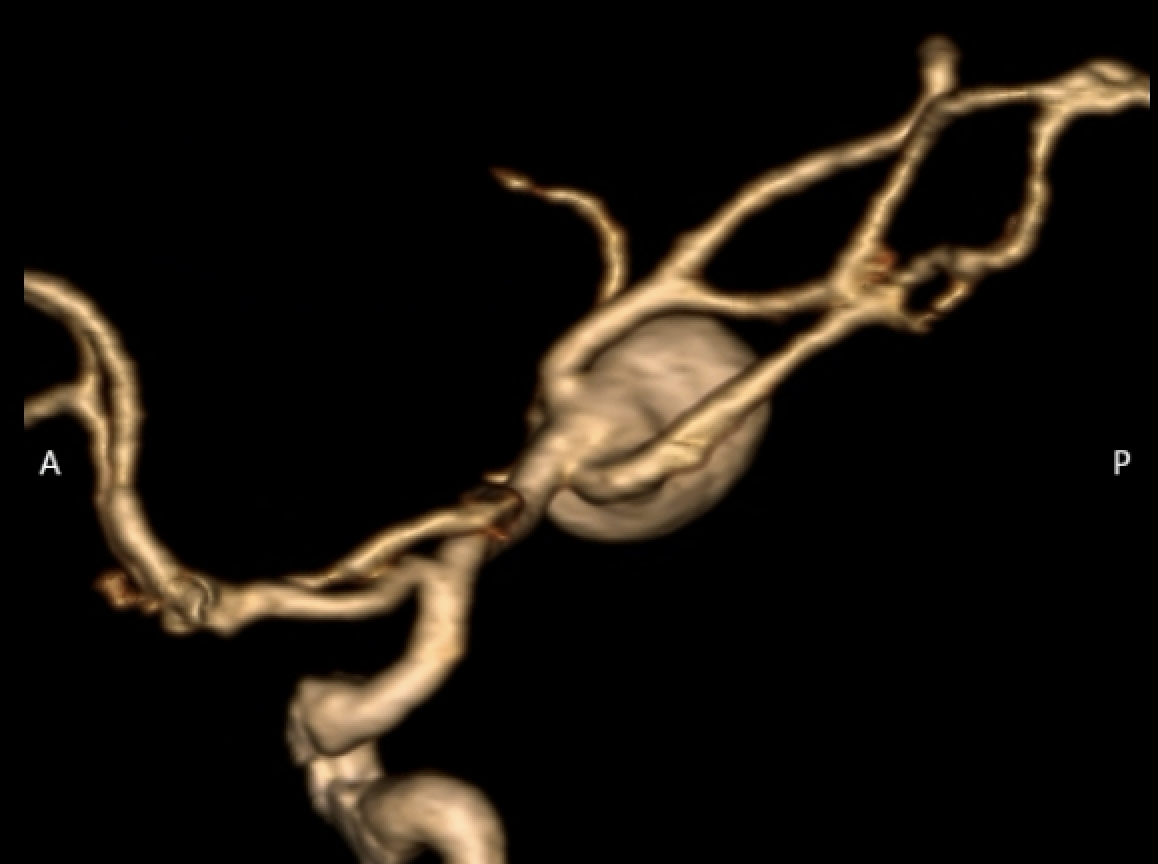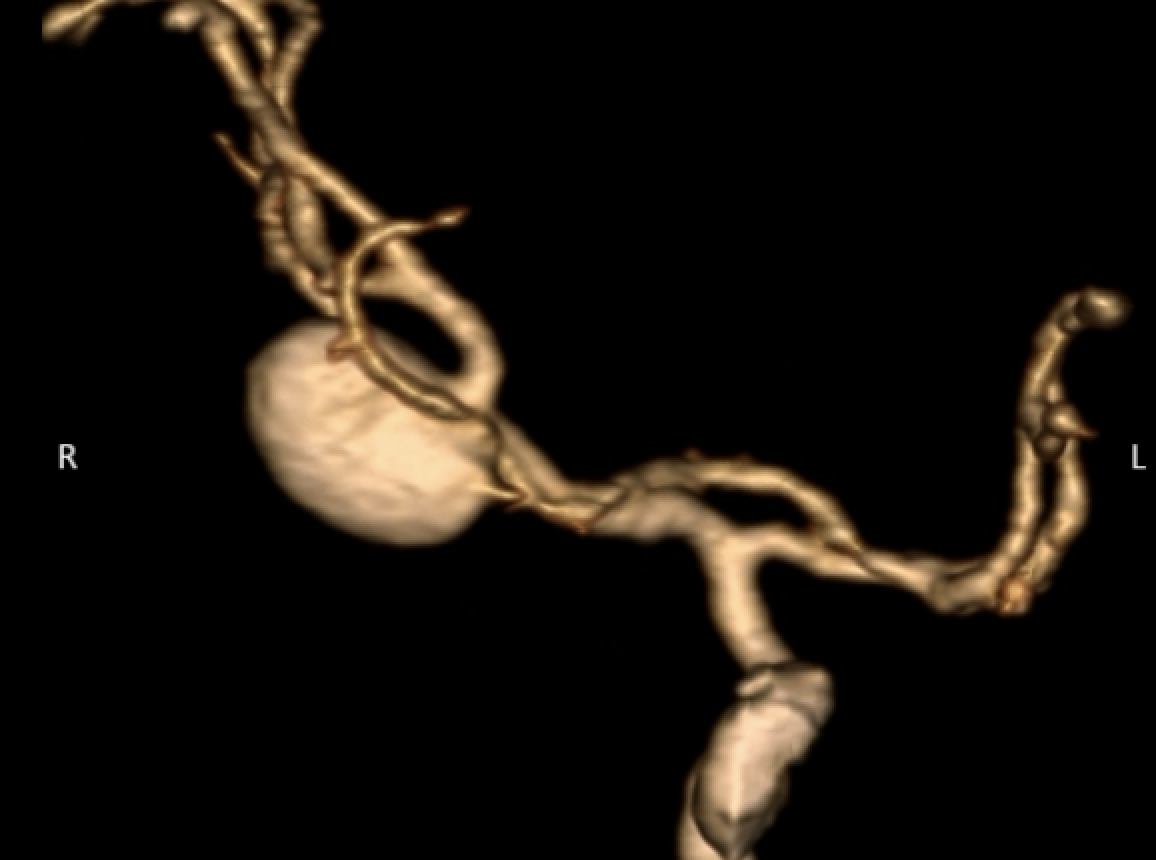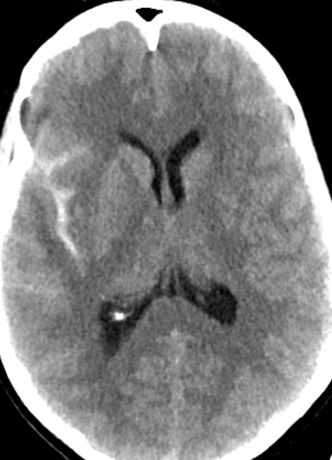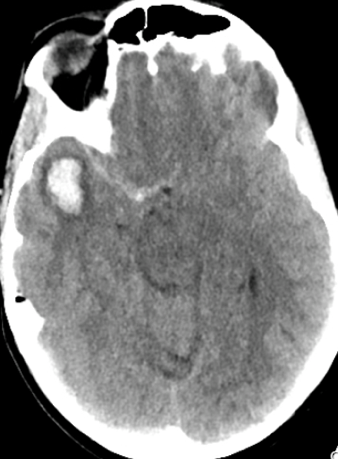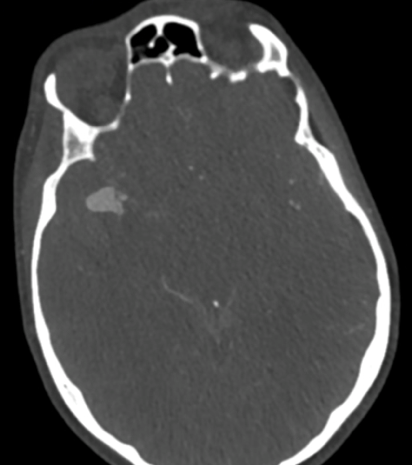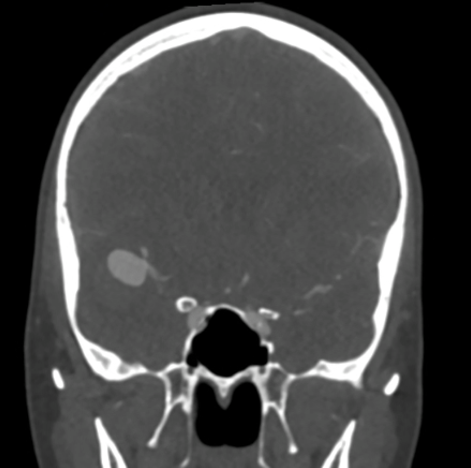  **Case 15** |

^a^No DSA or 3D imaging available and therefore not presented during the multidisciplinary meeting

*Abbreviations: A, anterior; DSA, digital subtraction angiography; L, left; MDT, multidisciplinary team, P, posterior; R, Right; 3D, three dimensional*

**Supplemental Table 2 | Composition of each multidisciplinary team**

| Multidisciplinary team characteristics |  |
| --- | --- |
| **Site 1** |  |
| No. of clinicians | 5 |
| No. of clinicians per specialty |  |
| - Neurosurgery | 3 |
| - Neurology | 0 |
| - Interventional-Radiology | 2 |
| - Interventional-Neurology | 0 |
| Male | 5 (100%) |
| Age, median (range) | 53 (36 – 62) |
| Years of post-residency experience, median (range) | 20 (0.3 – 25) |
|  |  |
| **Site 2** |  |
| No. of clinicians | 3 |
| No. of clinicians per specialty |  |
| - Neurosurgery | 1 |
| - Neurology | 1 |
| - Interventional-Radiology | 0 |
| - Interventional-Neurology | 1 |
| Male | 3 (100%) |
| Age, median (range) | 44 (35 – 52) |
| Years of post-residency experience, median (range) | 11 (1 – 17) |
|  |  |
| **Site 3*^a^*** |  |
| No. of clinicians | 2 |
| No. of clinicians per specialty |  |
| - Neurosurgery | 2 |
| - Neurology | 0 |
| - Interventional-Radiology | 0 |
| - Interventional-Neurology | 0 |
| Male | 2 (100%) |
| Age, median (range) | 58 (51 – 65) |
| Years of post-residency experience, median (range) | 25^b^ (16 – 33) |
|  |  |
| **Site 4** |  |
| No. of clinicians | 3 |
| No. of clinicians per specialty |  |
| - Neurosurgery | 1 |
| - Neurology | 1 |
| - Interventional-Radiology | 1 |
| - Interventional-Neurology | 0 |
| Male | 2 (67%) |
| Age, median (range) | 38 [36 – 42] |
| Years of post-residency experience, median (range) | 8 [6 – 8] |
|  |  |
| **Site 5** |  |
| No. of clinicians | 3 |
| No. of clinicians per specialty |  |
| - Neurosurgery | 1 |
| - Neurology | 1 |
| - Interventional-Radiology | 1 |
| - Interventional-Neurology | 0 |
| Male | 2 (67%) |
| Age, median (range) | 49 [45 – 49] |
| Years of post-residency experience, median (range) | 17 [11 – 20] |

*Abbreviations: No, number*

^a^In Site 3, all aneurysmal subarachnoid hemorrhage care is led by neurosurgery. Care is organized so treatment decision-making and performing primary treatment modalities befalls fully on the neurosurgery department.

^b^Rounded
